# Incidence of haematological malignancy by sub-type: a report from the Haematological Malignancy Research Network

**DOI:** 10.1038/bjc.2011.450

**Published:** 2011-11-01

**Authors:** A Smith, D Howell, R Patmore, A Jack, E Roman

**Affiliations:** 1 Epidemiology and Genetics Unit, Department of Health Sciences, University of York, York, UK; 2 Queens Centre for Oncology and Haematology, Castle Hill Hospital, Hull, UK; 3 Haematological Malignancy Diagnostic Service, St James's Institute of Oncology, Bexley Wing, St James's University Hospital, Leeds, UK; 4 Hull York Medical School, Heslington, York, UK

**Keywords:** descriptive epidemiology, incidence, lymphoma, leukaemia, myeloma, socio-economic status

## Abstract

**Background:**

Ascertainment of cases and disease classification is an acknowledged problem for epidemiological research into haematological malignancies.

**Methods:**

The Haematological Malignancy Research Network comprises an ongoing population-based patient cohort. All diagnoses (paediatric and adult) across two UK Cancer Networks (population 3.6 million, >2000 diagnoses annually, socio-demographically representative of the UK) are made by an integrated haematopathology laboratory. Diagnostics, prognostics, and treatment are recorded to clinical trial standards, and socio-demographic measures are routinely obtained.

**Results:**

A total of 10 729 haematological malignancies (myeloid=2706, lymphoid=8023) were diagnosed over the 5 years, that is, from 2004 to 2009. Descriptive data (age, sex, and deprivation), sex-specific age-standardised (European population) rates, and estimated UK frequencies are presented for 24 sub-types. The age of patients ranged from 4 weeks to 99 years (median 70.6 years), and the male rate was more than double the female rate for several myeloid and lymphoid sub-types, this difference being evident in both children and adults. No relationship with deprivation was detected.

**Conclusion:**

Accurate population-based data on haematological malignancies can be collected to the standard required to deliver reproducible results that can be extrapolated to national populations. Our analyses emphasise the importance of gender and age as disease determinants, and suggest that aetiological investigations that focus on socio-economic factors are unlikely to be rewarding.

To originate and test hypotheses about pathogenesis, it is vitally important to accurately describe the underlying disease patterns and trends (Parkin, 2006; Boyle, 2008; Ferlay *et al*, 2010). This requires complete ascertainment of cases within a defined population, as well as the application of appropriate disease classifications, and it is the attainment of these two key components that continues to beleaguer epidemiological research into the haematological malignancies (National Institute for Clinical Excellence, 2003; Sant *et al*, 2009, 2010).

Traditionally, the descriptive epidemiology of haematological malignancies considers four broad categories – leukaemia, Hodgkin lymphoma, non-Hodgkin lymphoma, and myeloma; with national and global organisations including the USA's Surveillance Epidemiology and End Results Program (www.seer.cancer.gov), the UK's National Cancer Intelligence Network (www.ncin.org.uk), and the World Health Organization (WHO)'s International Agency for Research on Cancer (http://globocan.iarc.fr/), routinely publishing data in this format (National Cancer Intelligence Network, 2008; Westlake, 2009; Jemal *et al*, 2010, 2011). Such counts show that, as a group, haematological neoplasms are comparatively common, accounting for around 9% of all cancers and being the fourth most frequently diagnosed cancer in both men (after prostate, lung, and colorectum) and women (after breast, lung, and colorectum) in economically developed regions of the world. However, over and above basic tallies, the usefulness of these descriptive data for epidemiological research is constrained by the classification system applied, which for haematological malignancies is largely rooted in the gradual recognition of disease entities at the beginning of the twentieth century. In the 1980s and 1990s, however, several competing classifications emerged as understanding about the relationship between the various haematological malignancies, the bone marrow, and the immune system, and the cellular and genetic basis of malignant transformation gradually increased. In the early 1980s, for example, the Working Formulation, which was developed as a method of translating between the many competing lymphoid classifications, rapidly became the standard in North America, and many epidemiological studies conducted there were based on this system. At the same time, the majority of European centres used the Kiel classification, making effective comparison of results between North America and Europe almost impossible (Harris *et al*, 1994).

In 2001, the WHO produced, for the first time, a consensus classification that defined all haematological malignancies in terms of immunophenotype, genetic abnormalities and clinical features (World Health Organization, 2001), and this is incorporated into the current version of the International Classification of Diseases for Oncology (ICD-O3); (Fritz, 2000). The strategy adopted was based on the principle devised for the Revised European–American Classification of Lymphoid Neoplasms, which was introduced in the mid-1990s (Harris *et al*, 1994). Unfortunately, however, although the new WHO classification and its successor (WHO, 2008) were adopted into clinical practice almost uniformly around the world, there was no immediate effect on population-based cancer information systems, where the practice of grouping haematological malignancies into the four broad groups defined in the tenth revision of ICD-10 (www.who.int/classifications/icd/en/) has tended to continue (National Cancer Intelligence Network, 2008; Westlake, 2009; Ferlay *et al*, 2010; Jemal *et al*, 2010, 2011). This is largely because unlike many other cancers, haematological neoplasms are diagnosed using multiple parameters, including a combination of histology, cytology, immunophenotyping, cytogenetics, imaging, and clinical data. This range and depth of data is difficult for cancer registries and other researchers to access systematically, potentially forming a barrier not only to the collection of diagnostic data at the level of detail required to systematically implement the latest WHO classification (WHO, 2001; WHO, 2008), but also to complete ascertainment (Sant *et al*, 2010). In recognition of this fact, a number of methods have been applied in an attempt to generate more informative descriptive data, including, for example, the application of bridge-coding algorithms to historically coded data (Sant *et al*, 2010; Turner *et al*, 2010; Maynadié *et al*, 2011) and the reporting of specialist hospital-based case-series frequencies (Yoon *et al*, 2010; Mozaheb *et al*, 2011). Inevitably, however, the accuracy and completeness of data generated by such initiatives has continued to pose serious interpretative problems for both researchers and health service planners.

In response to these challenges, the Haematological Malignancy Research Network (HMRN) was established in the UK in 2004 (Smith *et al*, 2010). HMRN is predicated on the framework of the UK National Health Service, where 37 cancer networks are responsible for bringing together health service commissioners and providers, the voluntary sector and local authorities to deliver high quality cancer care. HMRN presently covers two such cancer networks (Figure 1A), which comprise a single clinical network (population 3.6 million, with over 2000 new haematological neoplasms diagnosed each year), and the present report examines the socio-demographic characteristics of patients diagnosed over the first 5 years of the project, that is from 1 September 2004 to 31 August 2009.

## Materials and methods

Haematological Malignancy Research Network (www.hmrn.org) is an ongoing population-based cohort of patients (adult and paediatric) newly diagnosed with a haematological malignancy. It is a unique venture, combining the expertise of a single integrated haematopathology laboratory, a unified clinical network (comprising the Yorkshire and Humber and Yorkshire Coast Cancer Networks), and a specialist epidemiology unit, and full details of its structure, data-collection methods, and ethical approvals have been described in detail elsewhere (Smith *et al*, 2010). Briefly, as a matter of policy, all diagnoses within the clinical network are made and coded by clinical specialists to the latest WHO classification at a single integrated haematopathology laboratory – the Haematological Malignancy Diagnostic Service (www.HMDS.info) – which was cited in the UK Department of Health's Cancer Reform Strategy as ‘the model for delivery of complex diagnostic services’. Following diagnosis, and with an emphasis on obtaining primary-source data, information is abstracted from medical records and laboratory reports to clinical trial standards, and all diagnostic, prognostic, treatment, and outcome data are linked and held in a central database.

Populations and area-based measures of urban/rural status, and deprivation are routinely obtained from the UK census and other national data sources (Office for National Statistics, 2001; ONS Geography, 2004). For the purposes of the present report, subjects were given a measure of area-based deprivation assigned on the lower super output area, where they were resident at the time of diagnosis. In common with other reports (Shack *et al*, 2008; National Cancer Intelligence Network, 2009; Department of Health, 2010), the income domain of the index of multiple deprivation (IMD) was used (quintile one containing the most affluent fifth of England's lower super output areas, and quintile five the least). All analyses were conducted in the statistical package STATA 11. (Stata-Corp., 2010) Incidence rates, sex rate ratios, and 95% confidence intervals (CIs) were estimated by Poisson regression; directly age-standardised rates were calculated using the Stata command dstdize, and indirectly standardised-incidence ratios (SIR) were calculated using the Stata command istdize.

Descriptive findings are presented here for 10 729 haematological malignancies diagnosed within the HMRN region over 5 years spanning September 2004 to August 2009. For analytical purposes, these diagnoses coded to ICD-O3 are grouped into 24 main WHO categories; the codes that comprise these groups are published on our website and in Supplementary Table 1 (www.hmrn.org/Info/Disease_Classification.aspx).

## Results

**Figure 1:**
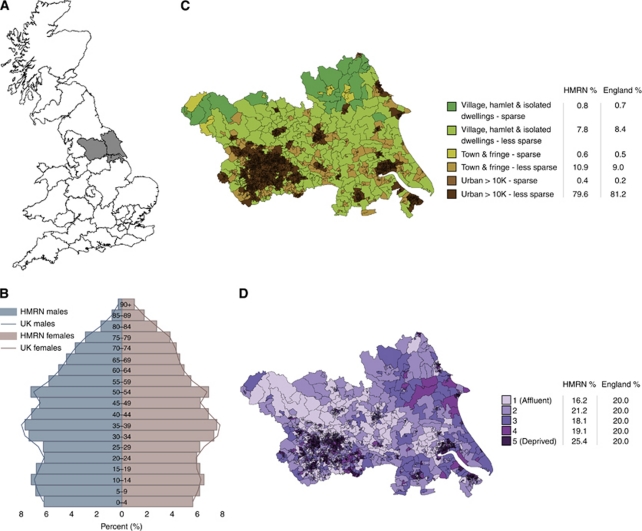
Socio-demographic structure of Haematological Malignancy Research Network (HMRN). (**A**) Map of study area. (**B**) Population, age, and sex structure. (**C**) Office for National Statistics urban/rural definition. (**D**) Index of multiple deprivation – income domain.

With a combined population of around 3.6 million, the socio-demographic structure of HMRN is broadly representative of the national population in terms of age, sex, urban/rural status, and deprivation (Figure 1). The 2001 age–sex distribution is compared with the UK (58.8 million) in Figure 1B, but in line with national data release policies, the urban/rural and deprivation configurations are compared with England alone (49.1 million) in Figure 1C and 1D, respectively. Although the age/sex distributions (Figure 1B) and urban/rural residence patterns (Figure 1C) closely mirror those of the national population, the HMRN region contains proportionately more people living in areas classified as deprived and fewer in areas classified as affluent (Figure 1D).

**Figure 2:**
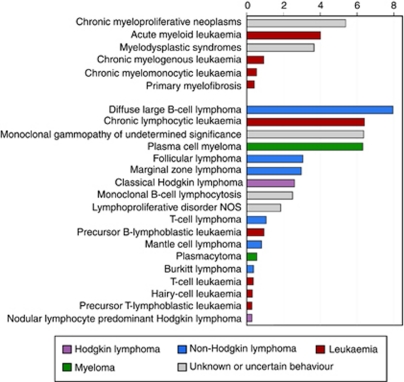
Annual crude rates per 100 000: Haematological Malignancy Research Network (HMRN), 2004–2009.

The 10 729 haematological malignancies diagnosed over the 5 years from September 2004 to August 2009 are distributed by sub-type in Table 1. Myeloid malignancies, which comprise around a quarter of the total (*N*=2706) are presented first, and lymphoid, which account for the remaining malignancies (*N*=8023), are second. Data on median ages at the time of diagnosis, annual rates, and sex-rate ratios (male rate/female rate) with 95% CIs are also given in Table 1. The rates in Table 1 are ordered by magnitude in Figure 2, and the bars are colour coded, differentiating the traditional groupings of leukaemia, non-Hodgkin lymphoma, Hodgkin lymphoma, and myeloma from other haematological neoplasms that are less consistently registered by national schemes. The classic ICD-10 leukaemia group contains a mix of myeloid and lymphoid conditions, the latter including both precursor and mature B-cell and T-cell subtypes. By contrast, within the traditional lymphoma and myeloma groupings, there is less diversity in the cell type of origin; with mature B-cell malignancies dominating. Indeed, with an annual rate of 7.9 per 100 000 per year, diffuse large B-cell lymphoma is the most common haematological malignancy, and chronic lymphocytic leukaemia (CLL), which like diffuse large B-cell lymphoma is also a mature B-cell neoplasm, is the next most common.

As detailed in the Introduction, the classification of haematological malignancies has changed markedly in recent decades, with several conditions once classified as neoplasms of uncertain or unknown behaviour now being categorised as malignant, and other conditions being recognised as part of the cancer continuum for the first time; the disorders falling into this category are shaded grey in Figure 2. Chronic myeloproliferative neoplasms and myelodysplastic syndromes now comprise around two-thirds of the myeloid neoplasms assigned a behaviour code of 3 (malignant primary site) in the WHO ICD-O3. Within the lymphoid category, lymphoproliferative disorders not otherwise specified also contains a mix of malignancies, all with behaviour codes of 3. However, although monoclonal gammopathy of undetermined significance (MGUS) and monoclonal B-cell lymphocytosis (MBL) are both conditions in which neoplastic B-cells are detectable, the diagnostic criteria for MBL being where the peripheral blood B-cell count is less than 5 × 10^9^/l lymphocytes (and in which risks of progression to myeloma in the case of the former and CLL in the case of the latter are elevated), their behaviour remains uncertain.

**Figure 3:**
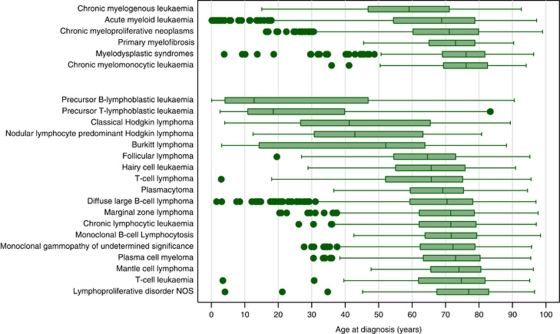
Age at diagnosis distributions: Haematological Malignancy Research Network (HMRN), 2004–2009.

As with most other cancers, the likelihood of being diagnosed with a haematological malignancy increases markedly with age, the median age at diagnosis being 70.6 years within the HMRN region (Table 1). However, unlike many other common cancers, haematological malignancy can be diagnosed at any age, with different subtypes dominating at different ages. More information about the age distributions of the various subtypes is presented in Figure 3, which shows box-and-whisker (boxplots) summary age plots ordered by the magnitude of the median for myeloid and lymphoid malignancies separately. The interquartile range is represented by the box, with outliers occurring outside the maximum data series of 1.5 times the interquartile range being shown as separate points.

The majority of myeloid conditions are diagnosed above 70 years of age, but sporadic cases arise at younger ages (Figure 3). Likewise, lymphoid malignancies generally occur in older people, but nonetheless span the entire age range, our youngest diagnosis having been made at 4 weeks of age and oldest at 99 years. The precursor B-cell and T-cell lymphoblastic leukaemias tend to occur at the youngest ages, the medians being 12.7 years and 18.5 years, respectively (Table 1). However, as with some of the conditions that principally occur at older ages, such as diffuse large B-cell lymphoma, these too are periodically diagnosed outside their normal age range. Such wide age spans are however not seen for all lymphoid conditions, including the rarer forms like hairy-cell leukaemia and mantle-cell lymphoma, and also comparatively common conditions like CLL and myeloma – all of which seldom, if ever, occur below the age of 30 years. A further conspicuous feature of lymphoid neoplasms is the similarity in the age distributions of certain closely related conditions such as MBL (median 71.7 years) and CLL (median 71.6 years), as well as monoclonal gammopathy of underdetermined significance (median 72.2 years) and myeloma (median 73.0 years), lying adjacent to each other in Figure 3.

**Figure 4:**
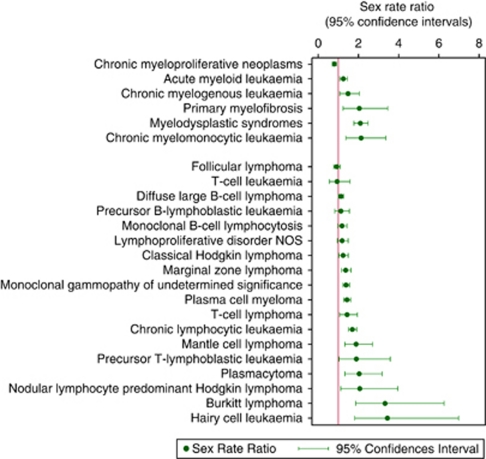
Sex-rate ratios: Haematological Malignancy Research Network (HMRN), 2004–2009.

**Figure 5:**
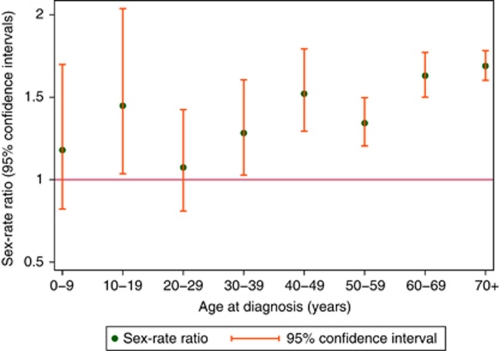
Sex-rate ratios by age: Haematological Malignancy Research Network (HMRN), 2004–2009.

In general, haematological malignancies tend to occur more frequently in males than females, and for many conditions, the rate among males is more than twice that of females (Table 1). The consistency of the gender difference is plainly visible in Figure 4, which shows the sex-specific rate ratios (male rate/female rate) ordered by magnitude. Indeed, conditions with no apparent sex bias, such as the chronic myleoproliferative neoplasms (male rate/female rate=0.80, 95% CI 0.70–0.91) and follicular lymphoma (male rate/female rate=0.92, 95% CI 0.77–1.09), stand out from the rest (Table 1). The lymphoid group exhibits some of the most striking sex differences, the rates of the comparatively rare Burkitt lymphoma and hairy-cell leukaemia being more than three times higher in males than in females. These sex differences occur across the full age spectrum, being seen in conditions with comparatively low, as well as high, median ages at diagnosis such as mantle-cell lymphoma (median age at diagnosis 74 years) and precursor T-lymphoblastic leukaemia (median age at diagnosis 18.5 years), for example, both with ratios approaching 2.0 lying adjacent to each other in Figure 4. The consistency of the gender bias is further illustrated in Figure 5, which shows the sex-rate ratios plotted in 10-year age groups for all haematological malignancies combined; the point estimates at younger ages being similar to those at older ages, although the CIs are wider, reflecting the comparative sparsity of the data.

**Figure 6:**
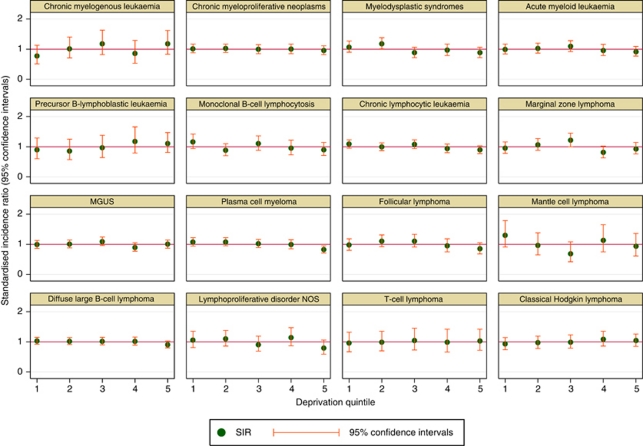
Standardised-incidence ratios (SIR) by index of multiple deprivation (IMD) income domain.

In the 24 main diagnostic categories listed in Table 1, 16 had 100 diagnoses or more, and for these the SIRs and 95% CIs are plotted by index of multiple deprivation income domain quintile in Figure 6, (group 1 being the most affluent and group 5 being the most deprived). No trends with deprivation are evident, although for some malignancies there is an indication of a deficit in the most deprived quintile; the most notable being myeloma where the SIR in category 5 is significantly below 1.0 (0.82 (95% CI 0.71–0.95).

The lack of a trend with deprivation (Figure 6) is particularly pertinent to precursor B-lymphoblastic leukaemia and classical Hodgkin lymphoma (CHL), both of which have been suggested to be increasingly common in more affluent families and communities. B-lymphoblastic leukaemia is primarily paediatric (Figure 3), and it is in this age group that an association with socio-economic status has been suggested. In our data, however, the results were similar when the analysis was restricted to cases diagnosed before the age of 15 years (95 out of 167); SIRs (95% CIs) for deprivation categories 1 through 5, respectively, being 0.7 (0.4–1.3), 1.0 (0.6–1.6), 1.1 (0.6–1.7), 1.0 (0.5–1.6), and 1.2 (0.8–1.6). Likewise, for CHL, the strongest effects have been reported at younger ages where the nodular sclerosis form of CHL predominates. In our data, no associations with deprivation were observed, either for total CHL or for any of the CHL subtypes (data not shown).

The size and demographic similarity of HMRN's population to the general UK population (Figure 1) means that the HMRN's data can reasonably be extrapolated to the country as a whole. The estimated UK totals, calculated by applying HMRN's age-specific rates to the corresponding general population age strata are shown in Table 2. For the purposes of wider comparability, age-standardised rates (European population) are also given in Table 2; these rates are in general lower than the actual rates (Table 1), reflecting the fact that unlike the real population (Figure 1), the hypothetical standard has a younger age structure with no excess of females in the older age groups. For the sake of completeness, information on MBL and monoclonal gammopathy of undetermined significance are included in Table 2, but their data are excluded from the overall totals.

## Discussion

Our ability to calculate reliable incidence rates for clinically meaningful haematological malignancy subtypes is a fundamental key research achievement; the analyses revealing notable associations with both age and sex, contrasting somewhat starkly with the comparative lack of variation with area-based measures of deprivation. In addition, the size and representative nature of our study population mean that our data can be extrapolated to the UK as a whole, providing for the first time, national estimates for the main WHO-defined disease entities (WHO, 2008). Indeed, HMRN rates could be applied to any well-characterised population, generating estimated or expected frequencies, depending on the assumptions made.

Haematological Malignancy Research Network was established with the aim of providing robust data to inform epidemiological research and clinical practice, the project being predicated on a comprehensive population-based patient cohort. Within HMRN's population of 3.6 million, which comprises 6% of the UK's estimated total, over 2000 new haematological malignancies are diagnosed each year. All of these diagnoses – irrespective of the patient's age, treatment intent, or management within the National Health Service/private sector – are made and coded by clinical specialists working within a single integrated haematopathology laboratory (www.hmds.info). Critically, an HMDS diagnosis is a fundamental policy requirement of the clinical network, and without it, treatment cannot occur. Furthermore, although outside the remit of the current report, it is important to note the longitudinal nature of HMRN's data collection processes, which include the collection of full sequential diagnostic and treatment histories (with response and outcome recorded for all episodes), and linkage to death certificates (‘flagging’) in the national scheme. Haematological malignancies, unlike other cancers, are characterised by their ability to transform and progress, and this is yet another aspect that challenges cancer registries. For example, the present report is based on 10 729 diagnoses, but these relate to 10 306 people diagnosed with a haematological cancer for the first time, of whom 407 (3.9%) had a second haematological neoplasm diagnosed, either concurrently or because their disease progressed or transformed, and 16 (<1%) had a third diagnosis. Investigating the epidemiology of transformation and progression, as well as other outcomes, will be the subject of future reports.

Comparing patterns and trends is a general feature of most descriptive epidemiological reports; and although frequencies for most subtypes cannot be compared with national programmes, because data are not coded in the same way, we can nonetheless confirm that our incidence rates are in line with expectation for those few clinically evident conditions where comparisons can be made. For example, our acute leukaemia and Hodgkin lymphoma rates are broadly similar to the most recent estimates published by SEER (www.seer.cancer.gov) and Cancer Research UK (http://info.cancerresearchuk.org/cancerstats). Indeed, our annual UK incidence estimate of 1664 diagnoses for all Hodgkin lymphomas combined is almost identical to the UK 2007 cancer registration count of 1673 (Cancer Research UK, 2010). Such agreements are reassuring not only for HMRN, but also for the national registration scheme. Moreover, a recent collaboration between HMRN and the National Cancer Data Repository, comparing observed registrations in England 2004–2007 with numbers expected on the basis of HMRN rates, showed good agreement for the conditions that could be compared nationally and by Cancer Network/Registry (Oliver *et al*, 2011).

Additional comparisons with the few specialist registries and/or consortia that have attempted to generate more informative data by applying bridge-coding algorithms are less rewarding. In addition to problems associated with defining catchment populations, bridge coding is inevitably associated with unquantifiable levels of misclassification, and with large numbers of neoplasms being categorised as ‘unknown’. For example, a recent attempt to bridge-code data for haematological malignancies diagnosed during 2000–2002 across 44 European registries produced disease-specific estimates for some, but not all, of the groups presented in the current report (Sant *et al*, 2010). Discrepancies were particularly marked for the lymphoid neoplasms, where some of the estimates were almost halved; for example, the UK age-standardised (European) rate estimate for diffuse large B-cell lymphoma was 3.7 per 100 000, which compares poorly with the 6.3 (95% CI 6.1–6.6) per 100 000 estimated by HMRN. The low rate reported by EUROCARE may be explained by the relatively high rate of ‘unknown’ lymphoid neoplasms (4.8 per 100 000), demonstrating how challenging it can be to apply the WHO classification retrospectively. This differs from the present study in which all diagnoses are coded to the latest WHO classification by clinical staff making the diagnosis.

Within most national and regional populations, the incidence of certain cancers is commonly observed to vary systematically with socio-economic factors for reasons that are known to be related either to their aetiology or to the likelihood of their detection. In England as a whole, for example, the most recent analysis of cancer registration data showed that as area-based affluence increased the incidence of cancers such as lung, stomach, and cervix fell, whereas the incidence of cancers such as melanoma, breast, and prostate increased (National Cancer Intelligence Network, 2009); and on a smaller scale, similar associations have been reported by the Northern and Yorkshire Cancer Registry and Information Service, within which our study area (www.hmrn.org) is located (Northern and Yorkshire Cancer Registry and Information Service, 2004). However, in contrast to many other cancers, no such systematic trends have been observed in the UK haematological malignancy data (National Cancer Intelligence Network, 2009). Hence, in this regard, our findings are broadly consistent with the national data, as using the same deprivation measure; we failed to uncover evidence of any significant trends for the subtypes examined. However, although no significant trends with deprivation were found within the HMRN region, a statistically significant reduction in the most deprived quintile for myeloma was found; and this has similarly been reported in the national data (National Cancer Intelligence Network, 2009) The explanation for these findings are unclear, but could reflect socio-economic variations in the likelihood of a diagnosis being made, the symptoms of myeloma often extending back over several months, and perhaps even years, before diagnosis (Friese *et al*, 2009). Indeed, the intermittent and non-specific nature of the symptoms associated with the onset of several haematological malignancies including follicular and marginal zone lymphomas pose similar diagnostic problems (Allgar and Neal, 2005; Howell *et al*, 2006, 2008). Interestingly, these diseases also showed similar deprivation patterns to myeloma, although these were not statistically significant.

It has long been known that most myeloid and lymphoid neoplasms are more common in males than females (National Cancer Intelligence Network, 2008; WHO, 2008; Smith *et al*, 2010); a favoured justification for this being that men are more likely than women to be exposed to potentially carcinogenic occupational and environmental agents (Alexander *et al*, 2007a, 2007b). However, this seems an unlikely explanation for the patterns seen within our data, as the male excess is evident in children as well as adults, and no relationship with deprivation was detected. Interestingly, the subtypes with the largest male excesses – Burkitt lymphoma and hairy-cell leukaemia – are both characterised by specific genetic abnormalities (WHO, 2008); and as it seems highly unlikely that gender influences rates of mutation, other explanations, including gender-specific differences in immune system regulation (Fish, 2008) may well be involved.

In addition to differences with gender, haematological malignancies exhibit characteristic age patterns that could also provide aetiological clues. This is particularly so for the lymphoid malignancies, where three broad overlapping patterns are discernable. Precursor T- and B-cell malignancies are primarily diseases of children and young adults, with sporadic cases occurring at older ages. On the other hand, malignancies arising from mature immunocompetent cells (mostly B lineage) predominate in adults, with sporadic cases of some, but not all, subtypes occurring at younger ages. Finally, a few disorders – notably the Hodgkin and Burkitt lymphomas – have more complex bimodal age distributions. All of these lymphoid neoplasms exhibit characteristic, but different, genetic abnormalities, and it would seem unlikely that the probability of any one individual mutation would be related directly to age. A more likely explanation is that the variations with age reflect the varying proportions of cell populations across the age range, with an immune system rich in precursor cells in young people and a predominance of germinal centre and memory B-cells in older adults.

The publication of the WHO classification of haematological malignancies was groundbreaking in that an international consensus was finally achieved. From an epidemiological perspective, it was a major advance, as it stressed the unity of the haematological malignancies as a group, emphasising the links between them, and in doing so, highlighting some of the arbitrary distinctions that had previously been made in many epidemiological studies – small lymphocytic lymphoma and CLL, for example. Furthermore, the WHO classification unequivocally recognised several entities as malignant disorders that are categorised as benign/uncertain in ICD-10: myelodysplastic syndromes for example, being placed within the broad spectrum of myeloid malignancies that includes acute myeloid leukaemia. The data presented in the report clearly show that such additions have a major impact on estimates of the overall disease burden, particularly in the myeloid group, in which the incidence is more than double. For epidemiology, it is equally important to recognise that the rapid rate of progress in understanding tumour biology, and the introduction of new diagnostic technologies and treatments mean WHO classifications, will inevitably require ongoing revision (World Health Organization, 2001; WHO, 2008; Jaffe, 2009; Vardiman *et al*, 2009; Campo *et al*, 2011). Fortunately for the present study, the Haematological Malignancy Diagnostic Service (www.hmds.info) which is at the heart of HMRN, is at the forefront of these developments; and such changes are incorporated as they occur. It seems highly unlikely, however, that these new technologies and concepts will be adopted in a uniform and timely fashion across all centres and countries; and hence, in the future, extrapolating data from initiatives such as HMRN may prove to be the best way of generating reliable information on haematological malignancies.

In conclusion, we have demonstrated that accurate population-based data collection for the whole range of haematological malignancies is achievable, and that this can be done across a sufficiently large and diverse area to deliver reproducible data that can be extrapolated to national populations. Our analyses emphasise the importance of gender and age as disease determinants, and suggest that aetiological investigations that focus on socio-economic factors are unlikely to be rewarding.

## Figures and Tables

**Table 1:** Numbers and annual crude rates per 100 000 (95% CI), median ages at diagnosis: Haematological Malignancy Research Network (HMRN), 2004–2009

	**Total**	**Median age at diagnosis**	**Annual rates (95% CI) per 100 000**			**Sex-rate ratio: male/female**
**Neoplasm (common abbreviation/synonym)**	**diagnoses**	**(years)**	**Total**	**Males**	**Females**	**(95% CI)**
*All diagnoses*	10 729	70.6	60.1 (58.9–61.2)	68.5 (66.8–70.3)	52.1(50.7–53.6)	1.31 (1.26–1.36)
						
*Total myeloid*	2706	72.0	15.1 (14.6–15.7)	16.9 (16.0–17.8)	13.5 (12.8–14.3)	1.25 (1.16–1.35)
Chronic myelogenous leukaemia (CML)	165	59.1	0.9 (0.8–1.1)	1.1 (0.9–1.4)	0.7 (0.6–0.9)	1.48 (1.07–2.05)
Primary myelofibrosis	73	73.0	0.4 (0.3–0.5)	0.6 (0.4–0.7)	0.3 (0.2–0.4)	2.04 (1.24–3.46)
Chronic myeloproliferative neoplasms (MPN)	961	71.1	5.4 (5.0–5.7)	4.8 (4.3–5.3)	6.0 (5.5–6.5)	0.80 (0.70–0.91)
Chronic myelomonocytic leukaemia (CMML)	96	76.1	0.6 (0.5–0.7)	0.8 (0.6–1.0)	0.4 (0.3–0.5)	2.10 (1.38–3.25)
Myelodysplastic syndromes (MDS)	653	76.1	3.7 (3.4–3.9)	5.0 (4.5–5.5)	2.4 (2.1–2.7)	2.09 (1.78–2.48)
Acute myeloid leukaemias (AML)	717	68.7	4.0 (3.7–4.3)	4.5 (4.0–4.9)	3.6 (3.2–4.0)	1.25 (1.07–1.45)
						
*Total lymphoid*	8023	70.1	44.9 (44.0–45.9)	51.6 (50.1–53.2)	38.6 (37.4–39.9)	1.34 (1.28–1.40)
Precursor B-lymphoblastic leukaemia (B-ALL)	167	12.7	0.9 (0.8–1.1)	1.0 (0.8–1.2)	0.9 (0.7–1.1)	1.13 (0.82–1.55)
Precursor T-lymphoblastic leukaemia (T-ALL)	50	18.5	0.3 (0.2–0.4)	0.4 (0.4–0.5)	0.2 (0.1–0.3)	1.89 (1.03–3.58)
Monoclonal B-cell lymphocytosis (MBL)	445	71.7	2.5 (2.3–2.7)	2.7 (2.4–3.1)	2.3 (2.0–2.6)	1.18 (0.98–1.43)
Chronic lymphocytic leukaemia (CLL)	1145	71.6	6.4 (6.0–6.8)	8.1 (7.5–8.7)	4.8 (4.4–5.3)	1.69 (1.50–1.91)
Marginal zone lymphomas (MZL)	530	71.5	3.0 (2.7–3.2)	3.4 (3.1–3.9)	2.5 (2.2–2.9)	1.37 (1.15–1.63)
Hairy-cell leukaemia (HCL)	55	65.7	0.3 (0.2–0.4)	0.5 (0.3–0.7)	0.1 (0.1–0.2)	3.44 (1.81–6.98)
Monoclonal gammopathy of undetermined significance (MGUS)	1135	72.2	6.4 (6.0–6.7)	7.4 (6.8–8.0)	5.4 (4.9–5.9)	1.38 (1.23–1.56)
Plasma cell myeloma (multiple myeloma)	1127	73.0	6.3 (5.9–6.7)	7.5 (6.9–8.1)	5.2 (4.8–5.7)	1.43 (1.27–1.61)
Plasmacytoma	99	69.2	0.6 (0.5–0.7)	0.8 (0.6–1.0)	0.4 (0.3–0.5)	2.03 (1.32–3.18)
Follicular lymphomas (FL)	547	64.6	3.1 (2.8–3.3)	2.9 (2.6–3.3)	3.2 (2.8–3.6)	0.92 (0.77–1.09)
Mantle-cell lymphoma	144	74.0	0.8 (0.7–0.9)	1.1 (0.9–1.3)	0.6 (0.4–0.7)	1.88 (1.33–2.70)
Diffuse large B-cell lymphomas (DLBCL)	1417	70.4	7.9 (7.5–8.4)	8.4 (7.8–9.1)	7.5 (6.9–8.1)	1.13 (1.01–1.26)
Burkitt lymphoma (BL)	66	52.2	0.4 (0.3–0.5)	0.6 (0.4–0.8)	0.2 (0.1–0.3)	3.33 (1.86–6.26)
Lymphoproliferative disorders NOS (LPD)	330	76.9	1.8 (1.7–2.3)	2.0 (1.7–2.3)	1.7 (1.4–2.0)	1.19 (0.95–1.87)
T-cell leukaemia	64	74.7	0.4 (0.3–0.5)	0.3 (0.2–0.5)	0.4 (0.3–0.5)	0.94 (0.56–1.58)
T-cell lymphoma	188	65.7	1.1 (0.9–1.2)	1.2 (1.0–1.5)	0.9 (0.7–1.1)	1.44 (1.07–1.94)
Nodular lymphocyte predominant Hodgkin lymphoma (NLPHL)	50	42.9	0.3 (0.2–0.4)	0.4 (0.4–0.5)	0.2 (0.1–0.3)	2.07 (1.12–3.95)
Classical Hodgkin lymphoma (CHL)	464	41.2	2.6 (2.4–2.8)	2.9 (2.5–3.3)	2.3 (2.0–2.7)	1.23 (1.02–1.49)

Abbreviations: CI=confidence interval; NOS=not otherwise specified.

**Table 2:** Estimated annual frequencies for the UK and European age-standardized rates per 100 000: based on HMRN sex- and age-specific rates data, 2004–2009

	**Estimated cases: UK**			**European age-standardised rate (95% CI)**		
**Neoplasm (common abbreviation/synonym)**	**Total**	**Males**	**Females**	**Total**	**Males**	**Females**
*All diagnoses* a	29 017^a^	16 264^a^	12 752^a^	40.8 (40.4–41.2)^a^	51.1 (50.4–51.7)^a^	32.7 (32.3–33.2)^a^
						
*Total myeloid*	8549	4693	3855	11.7 (11.5–11.9)	14.5 (14.1–14.8)	9.6 (9.3–9.9)
Chronic myelogenous leukaemia (CML)	533	313	220	0.8 (0.8–0.9)	1.0 (0.9–1.2)	0.7 (0.6–0.7)
Primary myelofibrosis	232	155	77	0.3 (0.3–0.4)	0.5 (0.4–0.6)	0.2 (0.1–0.2)
Chronic myeloproliferative neoplasms (MPN)	3138	1382	1756	4.3 (4.2–4.5)	4.4 (4.2–4.6)	4.4 (4.2–4.5)
Chronic myelomonocytic leukaemia (CMML)	300	204	96	0.4 (0.4–0.5)	0.6 (0.5–0.7)	0.2 (0.2–0.3)
Myelodysplastic syndromes (MDS)	2049	1382	667	2.5 (2.4–2.6)	4.0 (3.8–4.2)	1.5 (1.4–1.6)
Acute myeloid leukaemias (AML)	2275	1245	1029	3.2 (3.1–3.4)	4.0 (3.8–4.1)	2.7 (2.5–2.8)
						
*Total lymphoid* a	20 468^a^	11 571^a^	8897^a^	29.1 (28.8–29.5)^a^	36.6 (36.0–37.1)^a^	23.1 (22.7–23.5)^a^
Precursor B-lymphoblastic leukaemia (B-ALL)	540	279	261	1.0 (0.9–1.1)	1.1 (0.9–1.2)	1.0 (0.9–1.1)
Precursor T-lymphoblastic leukaemia (T-ALL)	159	102	57	0.3 (0.2–0.3)	0.4 (0.3–0.4)	0.2 (0.1–0.3)
Monoclonal B-cell lymphocytosis (MBL)^a^	1405^a^	752^a^	653^a^	1.9 (1.8–2.0)^a^	2.3 (2.2–2.5)^a^	1.6 (1.5–1.7)^a^
Chronic lymphocytic leukaemia (CLL)	3624	2259	1365	5.0 (4.8–5.1)	7.0 (6.8–7.2)	3.3 (3.1–3.4)
Marginal zone lymphomas (MZL)	1682	959	723	2.3 (2.2–2.4)	3.0 (2.8–3.1)	1.8 (1.7–1.9)
Hairy-cell leukaemia (HCL)	177	136	41	0.3 (0.2–0.3)	0.4 (0.4–0.5)	0.1 (0.1–0.2)
Monoclonal gammopathy of undetermined significance (MGUS)^a^	3601^a^	2059^a^	1542^a^	4.9 (4.8–5.0)^a^	6.3 (6.0–6.5)^a^	3.9 (3.7–4.1)^a^
Plasma cell myeloma (multiple myeloma)	3553	2073	1480	4.7 (4.6–4.9)	6.3 (6.1–6.6)	3.5 (3.4–3.7)
Plasmacytoma	318	211	107	0.5 (0.4–0.5)	0.7 (0.6–0.8)	0.3 (0.2–0.4)
Follicular lymphomas (FL)	1754	821	933	2.7 (2.6–2.8)	2.7 (2.5–2.8)	2.7 (2.5–2.8)
Mantle-cell lymphoma	454	295	159	0.6 (0.6–0.6)	0.9 (0.8–0.1)	0.4 (0.3–0.4)
Diffuse large B-cell lymphomas (DLBCL)	4502	2353	2149	6.3 (6.1–6.4)	7.3 (7.1–7.6)	5.5 (5.3–5.7)
Burkitt lymphoma (BL)	213	161	52	0.4 (0.3–0.4)	0.6 (0.5–0.7)	0.2 (0.1–0.2)
Lymphoproliferative disorders NOS (LPD)	1026	557	469	1.3 (1.2–1.4)	1.7 (1.6–1.8)	1.0 (0.9–1.1)
T-cell leukaemia	199	96	104	0.3 (0.2–0.3)	0.3 (0.2–0.4)	0.2 (0.2–0.3)
T-cell lymphoma	601	351	249	0.9 (0.8–1.0)	1.1 (1.0–1.3)	0.7 (0.6–0.8)
Nodular lymphocyte predominant Hodgkin lymphoma (NLPHL)	163	108	55	0.3 (0.2–0.3)	0.4 (0.3–0.5)	0.2 (0.1–0.2)
Classical Hodgkin lymphoma (CHL)	1501	809	692	2.5 (2.4–2.6)	2.8 (2.4–2.6)	2.2 (2.1–2.4)

Abbreviations: CI=confidence interval; NOS=not otherwise specified.

a Data for monoclonal B-cell lymphocytosis (MBL) and monoclonal gammopathy of undetermined significance (MGUS) are excluded from the totals.
