# Supplementary material for: Incidence of haematological malignancy by sub-type: a report from the Haematological Malignancy Research Network
Source: Br J Cancer. 2011 Nov 1;105(11):1684–92. doi: 10.1038/bjc.2011.450 (PMC3242607; doi:10.1038/bjc.2011.450)
Supplement: Supplementary Table 1 [file bjc2011450x1.doc]

Supplementary Table 1 ICD-O-3^1^ - HMRN 2004-9

| Lineage | Diagnosis | Subtype | ICD-O-3^1^ |
| --- | --- | --- | --- |
|  |  |  |  |
| Myeloid | Chronic myelogenous leukaemia | - | 9875/3 |
|  |  |  |  |
|  | Chronic myeloproliferative neoplasm^2^ | - | 9960/3 |
|  |  |  |  |
|  | Primary myelofibrosis | - | 9961/3 |
|  |  |  |  |
|  | Chronic myelomonocytic leukaemia | - | 9945/3 |
|  |  |  |  |
|  | Other myeloproliferative neoplasms | Systemic mastocytosis | 9741/3 |
|  |  | Chronic eosinophilic leukaemia | 9964/3 |
|  |  | Atypical chronic myeloid leukameia | 9876/3 |
|  |  | Juvenile chronic myelomonocytic leukaemia | 9946/3 |
|  |  |  |  |
|  | Myelodysplastic syndromes | Refractory anaemia with ringed sideroblasts | 9982/3 |
|  |  | Refractory anaemia with excess blasts | 9983/3 |
|  |  | Refractory cytopenia with multilineage dysplasia | 9985/3 |
|  |  | Myelodysplastic syndrome (5q-) | 9986/3 |
|  |  | Myelodysplastic syndrome, unclassifiable | 9989/3 |
|  |  |  |  |
|  | Acute myeloid leukaemia | Blastic plasmacytoid dendritic cell neoplasm | 9727/3 |
|  |  | AML NOS | 9861/3 |
|  |  | AML with NPM mutation as sole abnormality | 9861/3 |
|  |  | APML t(15;17)(q22;q11-12) | 9866/3 |
|  |  | AML with core binding factor | 9871/3 |
|  |  | AML with myelodysplasia-related changes | 9895/3 |
|  |  | AML with core binding factor | 9896/3 |
|  |  | AML with MLL (11q23) rearrangement | 9897/3 |
|  |  | AML - probable therapy related | 9920/3 |
|  |  |  |  |
| Lymphoid | Precursor B-Lymphoblastic Leukaemia | B-lymphoblastic leukaemia NOS | 9811/3 |
|  |  | B-lymphoblastic leukaemia with t(9;22) | 9812/3 |
|  |  | t(4;11)-associated acute leukaemia | 9813/3 |
|  |  | B-lymphoblastic leukaemia with t(12;21) | 9814/3 |
|  |  | B-lymphoblastic leukaemia with hyperdiploidy | 9815/3 |
|  |  | B-lymphoblastic leukaemia with hypodiploidy | 9816/3 |
|  |  |  |  |
|  | Monoclonal B-Cell Lymphocytosis | - | 9823/3 |
|  |  |  |  |
|  | Monoclonal Gammopathy of Undetermined Significance | - | 9765/1 |
|  |  |  |  |
|  | Chronic Lymphocytic Leukaemia | - | 9823/3 |
|  |  |  |  |
|  | Hairy Cell Leukaemia | - | 9940/3 |
|  |  |  |  |
|  | Plasma Cell Myeloma | - | 9732/3 |
|  |  |  |  |
|  | Plasmacytoma | Solitary plasmacytoma of bone | 9731/3 |
|  |  | Extraosseous plasmacytoma | 9734/3 |
|  |  |  |  |
|  | Marginal Zone Lymphoma | Extranodal marginal zone lymphoma | 9699/3 |
|  |  | Systemic marginal zone lymphoma | 9689/3 |
|  |  |  |  |
|  | Follicular Lymphoma | Follicular lymphoma | 9690/3 |
|  |  | Follicular lymphoma with large cell transformation | 9698/3 |
|  |  |  |  |
|  | Mantle Cell Lymphoma | - | 9673/3 |
|  |  |  |  |
|  | Diffuse Large B-Cell Lymphoma | B-cell Lymphoma, unclassifiable, with features intermediate between  Diffuse Large B-cell lymphoma and Hodgkin Lymphoma | 9596/3 |
|  |  | Mediastinal large B-cell lymphoma | 9679/3 |
|  |  | Diffuse large B-cell lymphoma, not otherwise specified | 9680/3 |
|  |  | T-cell/histiocyte-rich large B-cell lymphoma | 9688/3 |
|  |  | Intravascular large B-cell lymphoma | 9712/3 |
|  |  | Plasmablastic large B-cell lymphoma | 9735/3 |
|  |  | ALK-positive large B-cell lymphoma | 9737/3 |
|  |  |  |  |
|  | Lymphoproliferative disorders NOS (LPD) | - | 9591/3 |
|  |  | - | 9823/3 |
|  |  |  |  |
|  | Burkitt Lymphoma | - | 9687/3 |
|  |  |  |  |
|  | Precursor T-Lymphoblastic Leukaemia | - | 9837/3 |
|  |  |  |  |
|  | T-Cell Leukaemia | T-cell or NK cell large granular lymphocytosis | 9831/3 |
|  |  | T-cell prolymphocytic leukaemia | 9834/3 |
|  |  |  |  |
|  | T-Cell Lymphoma | Mycosis fungoides | 9700/3 |
|  |  | Sezary syndrome | 9701/3 |
|  |  | Peripheral T-cell lymphoma - common; unspecified | 9702/3 |
|  |  | Angioimmunoblastic T-cell lymphoma | 9705/3 |
|  |  | Anaplastic large cell lymphoma of T/null type | 9714/3 |
|  |  | Enteropathy-type T-cell lymphoma | 9717/3 |
|  |  | Primary cutaneous CD30 positive T-cell | 9718/3 |
|  |  | Extranodal NK/T-cell lymphoma, nasal type | 9719/3 |
|  |  | Adult T-cell lymphoma/leukaemia (HTLV-1 positive) | 9827/3 |
|  |  |  |  |
|  | Nodular Lymphocyte Predominant Hodgkin Lymphoma | - | 9659/3 |
|  |  |  |  |
|  | Classical Hodgkin Lymphoma | Hodgkin lymphoma - HIV associated | 9650/3 |
|  | Classical Hodgkin Lymphoma | Lymphocyte-rich classical Hodgkin lymphoma | 9651/3 |
|  | Classical Hodgkin Lymphoma | Mixed cellularity classical Hodgkin lymphoma | 9652/3 |
|  | Classical Hodgkin Lymphoma | Nodular sclerosis classical Hodgkin lymphoma | 9663/3 |

^1^ International Classification of Diseases for Oncology 3^rd^ Edition

^2^ Blood count data was not available at the time of diagnosis to the central diagnostic laboratory. Accordingly, the number of cases of essential thrombocythemia and polycythemia vera cannot be defined, likewise the diagnostic category MDS/MPN unclassifiable.
